# Supplementary material for: Physiotherapist beliefs and perspectives on virtual reality supported rehabilitation for the management of musculoskeletal shoulder pain: A focus group study
Source: PLoS One. 2023 Apr 14;18(4):e0284445. doi: 10.1371/journal.pone.0284445 (PMC10104293; doi:10.1371/journal.pone.0284445)
Supplement: S1 File — (DOCX) [file pone.0284445.s001.docx]

Focus Group Question Schedule

1. Did you enjoy the experience of using immersive virtual reality (VR)?
2. Did you experience any barriers in terms of usability of VR? Was it straightforward to set up?
3. What barriers do you expect would limit the use of VR in clinical practice? And how might these be overcome?
4. Did you experience any adverse effects during or after VR use?
5. Do you envisage a role for immersive VR in the assessment and rehabilitation of individuals with musculoskeletal shoulder pain?
6. If so, what aspect of VR do you think has the potential to be most beneficial? (Exercise/movement, distraction, engagement, immersion, motivation, competition, reward, education, exposure?)
7. If there is little engagement with question 7, consider asking specifically: what do you think about the use of VR for: Exercise/movement, distraction, engagement, immersion, motivation, competition, reward, education, exposure?
8. What specific subgroup of individuals with shoulder pain do you think would be most suited to using VR as part of their management? (Rotator Cuff Related Shoulder Pain, Frozen Shoulder, Glenohumeral Joint Osteoarthritis, Glenohumeral Joint Instability?)
9. Do you think there are any factors that might facilitate the use of VR in clinical practice?
