# Supplementary material for: Physiotherapist beliefs and perspectives on virtual reality supported rehabilitation for the management of musculoskeletal shoulder pain: A focus group study
Source: PLoS One. 2023 Apr 14;18(4):e0284445. doi: 10.1371/journal.pone.0284445 (PMC10104293; doi:10.1371/journal.pone.0284445)
Supplement: Attachment — Submitted filename: Response to Reviewers.docx [file pone.0284445.s003.docx]

Response to Reviewers

Reviewer 1

Dear Reviewer,

Thank you for your helpful insights and recommendations that will only help to strengthen the study. We hope that you will be satisfied with our revised version. Please see below for a detailed response to each point you have made:

| Line 1-2: The will of the authors to find a “captivating” title is understandable. However to quote the phrase “Within five seconds you can see the potential of this” in the title it seems to be overestimating the content of the article. In fact as the authors reported in lines 392-394 “Despite having reported that VR shows potential as an intervention for managing shoulder conditions, physiotherapists were slow to recommend VR in its current form and felt that further research and development would be needed before it could be incorporated into clinical practice.” | Thank you for your comment. We hoped to choose a quote that would reflect the essence of the study but understand what you say about this one potentially overstating the enthusiasm for VR. The quotation in the title has now been removed. |
| --- | --- |
| Line 117-120: In the introduction it does not seem relevant to report all the numerical results and p-values of cited studies. I suggest to remove them | Thank you. Numerical results have been removed. |
| Line 122-124: In the introduction it does not seem relevant to report all the numerical results and p-values of cited studies. I suggest to remove them | Thank you. Numerical results have been removed. |
| Line 126-131: In the introduction it does not seem relevant to report all the numerical results and p-values of cited studies. I suggest to remove them | Thank you. Numerical results have been removed. |
| Line 135-142: In the introduction it does not seem relevant to report all the numerical results and p-values of cited studies. I suggest to remove them | Thank you. Numerical results have been removed. |
| Line 139: In the cited study the p-value of the VR group was 0.027. If the authors want to report the rounded result it has to be 0.03 and not 0.02 | Thank you. Numerical results have been removed. |
| Line 140: There is not a space between (p=0.4) and (53). There is not the point mark after (53) Line 140: Reference 53 does not seem to be the correct one relative to the text | Thank you. Numerical results and the incorrect citation have been removed. |
| Line 145: Authors did not insert the reference number but just the word (ref) | Thank you for pointing this out. There is no reference for this statement, rather an expansion of the point in the following sentence. The word (ref) has been removed. |
| Line 173: There is not a space between “checklist” and “(59)” | Thank you. A space has been added here. |
| Line 203-204: “Participants were asked to use the headset at least three times for a maximum 20-minute duration in each session” Could the author provide a reference for this choice or at least motivate it? | Thank you. The Oculus Quest safety instructions advise using VR for a maximum 30-minute duration. We decided to recommend a 20-minute limit as all participants were new to the technology and we felt that this would minimise fatigue and potential motion sickness during a short trial period like this We felt that three 20-minute sessions would allow sufficient time to experience VR and engage with the focus group discussion. We have added justification in the paper’s text now. |
| Line 295: “Several” Provide the exact number of physiotherapists who have reported it | Thank you for your suggestion and we understand how you may value clarity on numbers of physiotherapists reporting perceptions.  On this matter, we believe that adding numbers of participants here may detract from the inductive process of the qualitative analysis. In a focus group interview, when a participant raises a point or opinion others may state their agreement or disagreement while others may engage by simply nodding in agreement. The fact that one person stated a point sometimes means that others may not feel the need to repeat it, but this is not necessarily reflective of their position regarding the point being made.  In addition, counting number of participants who stated or agreed with a point assumes that that the strength of feeling or perception (or whatever people are agreeing to) between each participant is the same if they ‘agree’. Some participants can feel very strongly about an issue, some will be carried along by group consensus and some will feel ambiguous, but all responses may get counted in the same pile. Our aim is to capture how the issues are much more nuanced as are participants perceptions. We believe that this is more in line with qualitative research practices. |
| Line 303: “A number” Provide the exact number of physiotherapists who have reported it | Thank you, we have included the specific number of participants here as it is a specific detail, not open to perception and we believe that it adds clarity to the story. |
| Line 334: “Most” Provide the exact number of physiotherapists who have reported it | As above, we believe that adding a specific number here does not add to the presentation of results and does not accurately reflect the strength of the discussion around this point of view. Our preference would be to not use numbers in this instance. |
| Line 354: “Most” Provide the exact number of physiotherapists who have reported it | As above, we feel that the use of numbers is not helpful here but have instead revised the wording of the sentence. |
| Line 363: “Some” Provide the exact number of physiotherapists who have reported it | As above, we feel that the use of numbers is not helpful here. In the discussion this point was intertwined with the discussion around using VR for improving motivation to exercise and therefore, it is difficult to separate the numbers of participants suggesting each opinion/perception regarding the use of VR in clinical practice. |
| Line 373: “Less commonly” Provide the exact number of physiotherapists who have reported it | Again, we prefer not to add specific numbers here but instead have revised the wording. |
| Line 383: There is not the quotation mark at the beginning of the phrase | Thank you. This has now been added. |
| Line 395: “risk or injury”; are “risk of injury” the correct words? | Thank you. This has now been corrected. |
| Line 432: “Some” Provide the exact number of physiotherapists who have reported it | Thank you. In this case we agree that including a number is appropriate as this is a specific finding reported by physiotherapists. |
| Line 540: There is not the bracket after 61% | Thank you. I have added a bracket here. |
| Line 555: Reference (30) does not seem to be the correct one relative to the text | Thank you. This was inserted by mistake. It has now been removed. |
| Line 556: Does not seem relevant to report the p-values of this cited study. I suggest to remove it | Thank you. Numerical results have been removed. |
| Line 560: Does not seem relevant to report the p-values of this cited study. I suggest to remove it | Thank you. Numerical results have been removed. |
| Line 870: “with question 7”; is it correct or it has to be “with question 6”? | Thank you. This has been corrected this to say “with question 6” |

Reviewer 2

Dear Reviewer,

Thank you for your kind words and helpful comments regarding our recent study. We hope that this study will help to further the development and evaluation of virtual reality rehabilitation for musculoskeletal shoulder pain in future years.

Yours Sincerely,

Niamh Brady
