# Supplementary material for: Physiotherapist beliefs and perspectives on virtual reality supported rehabilitation for the management of musculoskeletal shoulder pain: A focus group study
Source: PLoS One. 2023 Apr 14;18(4):e0284445. doi: 10.1371/journal.pone.0284445 (PMC10104293; doi:10.1371/journal.pone.0284445)
Supplement: Attachment — Submitted filename: Response to Reviewer.docx [file pone.0284445.s004.docx]

Response to Reviewer 1

Dear Reviewer,

Thank you for your helpful insights and recommendations. We hope that you will be satisfied with our revised version. Please see below for a detailed response to each point you have made:

| Line 118: There is not a space between “follow up” and “[46-48]” | Thank you for your comment. This has now been corrected. |
| --- | --- |
| Line 131: Still present the “(p<0.05)” I suggest to remove it. | Thank you. These numerical results have now been removed. |
| *“On this matter, we believe that adding numbers of participants here may detract from the inductive process of the qualitative analysis. In a focus group interview, when a participant raises a point or opinion others may state their agreement or disagreement while others may engage by simply nodding in agreement. The fact that one person stated a point sometimes means that others may not feel the need to repeat it, but this is not necessarily reflective of their position regarding the point being made. In addition, counting number of participants who stated or agreed with a point assumes that that the strength of feeling or perception (or whatever people are agreeing to) between each participant is the same if they ‘agree’. Some participants can feel very strongly about an issue, some will be carried along by group consensus and some will feel ambiguous, but all responses may get counted in the same pile. Our aim is to capture how the issues are much more nuanced as are participants perceptions. We believe that this is more in line with qualitative research practices.”*  I found the arguments of the authors correct and understandable. It would be helpful to somehow put these motivations into the discussion. | Thank you for your comment. We have now added a brief explanation of our approach to the results section and a more detailed discussion of our motivations to the discussion. |

Yours Sincerely,

Niamh Brady
