# Supplementary material for: Physiotherapist beliefs and perspectives on virtual reality supported rehabilitation for the management of musculoskeletal shoulder pain: A focus group study
Source: PLoS One. 2023 Apr 14;18(4):e0284445. doi: 10.1371/journal.pone.0284445 (PMC10104293; doi:10.1371/journal.pone.0284445)
Supplement: Attachment — Submitted filename: Response to Reviewer.docx [file pone.0284445.s005.docx]

Response to Reviewer 1

Dear Reviewer,

Thank you for reviewing the most recent draft of our manuscript with track changes. I failed to upload the correct version of the “Manuscript” and have now added the most recent version, without track changes. Thanks again for your input and helpful recommendations to date, which have certainly strengthened our paper.

Yours Sincerely,

Niamh Brady
